# Prevalence and Associated Risk Factors of Intestinal Parasites and Enteric Bacterial Infections among Selected Region Food Handlers of Ethiopia during 2014–2022: A Systematic Review and Meta-Analysis

**DOI:** 10.1155/2022/7786036

**Published:** 2022-10-12

**Authors:** Abayeneh Girma, Aleka Aemiro

**Affiliations:** Department of Biology, College of Natural and Computational Science, Mekdela Amba University, P.O. Box 32, Tuluawlia, Ethiopia

## Abstract

Food-borne disease due to intestinal parasites (IPs) and enteric bacterial infections (EBIs) remain a major public health problem. Food handlers, individuals involved in preparing and serving food, working with poor personal hygiene could pose a potential threat of spreading IPs and EBIs to the public. The aim of this study was to examine the overall prevalence and risk factors of IPs and EBIs among food handlers in four selected regions of Ethiopia. Scientific articles written in English were recovered from PubMed, ScienceDirect, Web of Science, Google Scholar, Cochrane Library, and other sources from Google Engine and University Library Databases. “Prevalence,” “Intestinal Parasites,” “Enteric Bacterial Infections,” “Associated Factors,” “Food Handlers,” and “Ethiopia” were the search terms used for this study. For critical appraisal, PRISMA 2009 was applied. Stata software version 16 was used to perform the meta-analysis. Heterogeneity and publication bias were evaluated using Cochran's *Q*, inverse variance (*I*^2^), and funnel plot asymmetry tests. A random-effects model was used to calculate the pooled burden of IPs and EBIs and its associated factors among food handlers, along with the parallel odds ratio (OR) and 95% confidence interval (CI). For this meta-analysis, a total of 5844 food handlers were included in the 20 eligible studies. The overall pooled prevalence of IPs and EBIs among food handlers in four selected regions of Ethiopia was 29.16% (95% CI: 22.61, 35.71), with covering (25.77%) and (3.39%) by IPs and EBIs, respectively. *Ascaris lumbricoides*, *Entamoeba histolytica*/*dispar*, *Giardia lamblia*, and hookworm were the most prevalent IPs among food handlers with a pooled prevalence of 7.58%, 6.78%, 3.67%, and 2.70%, respectively. *Salmonella* and *Shigella* spp. were the most prevalent EBIs among food handlers with a pooled prevalence of 2.78% and 0.61%, respectively. A high prevalence of IPs and EBIs among food handlers was observed in Oromia (38.56%; 95% CI: 29.98, 47.14), while a low prevalence was observed in the Tigray region (19.45%; 95% CI: 6.08, 32.82). Food handlers who had not taken food hygiene training (OR: 0.68, 95% CI: −0.34, 1.69), untrimmed finger nail (OR: 2.23, 95% CI: 1.47, 2.99), lack of periodic medical checkup (OR: 1.52, 95% CI: 0.41, 2.64), lack of handwashing habits (OR: 1.97, 95% CI: 0.53, 3.41), and eating raw vegetables and meat (OR: 2.63, 95% CI: 0.92, 4.34) were factors significantly associated with the prevalence of IPs and EBIs. The prevalence of IPs and EBIs was high in the selected Ethiopian region (Amhara, Oromia, SNNPR, and Tigray) food handlers along an increasing prevalence trend from 2014 to 2022. Therefore, this study recommends the provision of proper health education and training regarding personal hygiene, hand washing, food handling, medical checks, as well as raw vegetable and meat safety.

## 1. Introduction

Food-borne diseases are illnesses caused by ingesting pathogenic microorganisms (bacteria, fungi, viruses, and parasites) or their toxins (for bacteria and fungi) [1, 2]. Food-borne outbreaks can result in both health and economic losses. According to the World Health Organization (WHO) [3], 600 million people worldwide become severely ill each year, with 420,000 dying as a result of food contamination. Food-borne infections affect an estimated 48 million people in the United States each year, resulting in 128,000 hospitalizations and 3,000 fatalities [4, 5]. Food-borne and waterborne infections were also projected to cause approximately 700,000 deaths each year in Africa [6] and cause both short-term (nausea, vomiting, and diarrhea) and long-term (tissue damage, cancer, kidney or liver failure, brain disorders, and neural disorders) disorders.

Gastrointestinal parasitic infections are prevalent throughout the world, with the highest prevalence in poorer nations due to poor personal hygiene, environmental sanitation, socioeconomic, demographic, and health-related behaviors [7]. The most common way for intestinal parasitic infections to spread is through contaminated food and water, but they can further transmit from person-to-person through fecal-oral contact [8]. In the world, intestinal parasites infect approximately one-third of the total population, with the tropics and subtropics bearing the greatest load [9]. *Ascaris lumbricoides*, *Trichuris trichiura*, hookworm, *Entamoeba histolytica*, and *Giardia lamblia* infect an estimated 1.2 billion, 795 million, 740 million, 500 million, and 2.8 million people worldwide [10, 11]. In Ethiopia, the burden of intestinal parasites (IPs) is extremely high. A third (26 million), a quarter (21 million), and one in every eight (11 million) Ethiopians are infected with *Ascaris lumbricoides*, *Trichuris trichiura*, and hookworm [12], respectively. As a result, Ethiopia has the second, third, and fourth largest burdens of ascariasis, hookworm, and trichuriasis, in sub-Saharan Africa, respectively.

Enteric bacterial infection (EBI)-causing microbes, namely, the genus of *Salmonella* (Salmonellosis) and *Shigella* (Shigellosis) are also the most important sources of food-borne diseases. As a result, they continue to be significant public health issues. Furthermore, clinical prevention and control of typhoid fever are difficult, particularly in Africa, due to the development of antibiotic resistance, and vaccines are not immunogenic to young children. Globally, there are 93.8 million cases of gastroenteritis caused by *Salmonella* species each year, with 155,000 deaths. An estimated 80.3 million of these cases were food-borne [13]. *Shigella* species are more common in temperate and tropical areas. *Shigella* species causes an estimated 80–165 million cases of disease and 600,000 deaths worldwide each year [14, 15].

Food handlers are individuals engaged in preparing and serving foods, infected with gastrointestinal parasitic and bacterial infections, and practicing poor personal hygiene could be serious sources of transmission of IPs and EBIs to the public. Since food handlers infected with IPs and EBIs exhibit subclinical symptoms and are asymptomatic carriers, they are unaware of their possible role in the spread of infection, hinders the pathogens, and challenges in the integrated control and elimination of infections [8]. Furthermore, food handlers have a large impact on the spread of IPs and EBIs as they can directly or indirectly transmit infections to a large number of foods and drink consumers in food service establishments namely restaurants, hotels, factories, canteens, schools, hospitals, prisons, or other places where food is prepared and served to a range of users [16, 17].

Aside from socioeconomic issues, other factors including the availability of clean water, the survival of pathogenic parasites and bacteria in different environmental conditions, and personal and public hygiene habits all play an important role in the transmission of IPs and EBIs [18, 19]. Ethiopia has one of the lowest rates of clean water supply and toilet coverage [20]. Ethiopian studies on personal hygiene factors, for example, hand washing after toilet use, medical cheek examinations including stool exams, eating raw vegetables and meat, hand washing before food handling and meal, finger nail status, food hygiene training, and knowledge of enteric parasites and bacteria were found to contribute to the prevalence of IPs and EBIs among food handlers [12, 21, 22].

Numerous studies have been conducted in Africa, to investigate the prevalence of intestinal parasitic and enteric bacterial infections among handlers of food. In Ethiopia, the risk of *Salmonella* and *Shigella* infection among food handlers ranged from 1 to 7.5% [21, 23, 24]. However, the prevalence of intestinal parasitic infections among food handlers varied and was inconsistent across studies: 10.9 to 45.3% in Ethiopian university cafeterias [25–30], 43.9% in street dwellers [31], 48.1% in prisons [32], 35% in orphanage centers [33, 34], 32.3% in public hospitals [34], and 14.5 to 44% in restaurants and cafeterias [12, 22]. IPs and EBIs are one of the common public health issues in Ethiopia, with varying levels of prevalence throughout the country. In Ethiopia, only one systematic review and meta-analysis study were conducted on the prevalence of IPs among food handlers by Yimam et al. [12]. However, the prevalence and risk factors of both IPs and EBIs among selected region food handlers is not collected, well-organized, or recorded as a systematic review and meta-analysis. As a result, the objective of this study was to provide tangible evidence on the overall prevalence and risk factors for IPs and EBIs among food handlers using previously conducted research articles in four (Amhara, Oromia, SNNPR, and Tigray) regions of Ethiopia. Furthermore, the results obtained in the current investigation could significantly contribute to healthcare providers, users, and policy makers.

## 2. Methods

### 2.1. Profile of the Country

Ethiopia measures 1,104,300 square kilometers and is located in the Horn of Africa (total land area is 1,000,000 square kilometers) (386,102 square miles). Ethiopia is bordered in the north by Eritrea, in the east by Djibouti and Somalia, in the west by Sudan and South Sudan, and in the south by Kenya. According to Worldometer's elaboration of the most recent United Nations data, Ethiopia's current population was 113,881,451 in 2020, which is comparable to 1.47%. Furthermore, according to the aforementioned report, approximately, 21.3% of the population (24, 463, 423) will live in urban areas by 2020 [35, 36].

### 2.2. Search Strategy

**Figure 1:**
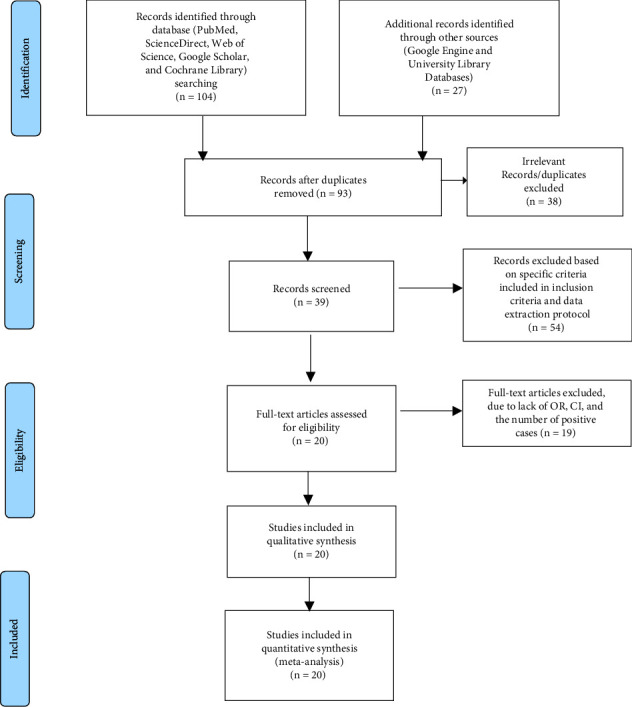
PRISMA 2009 flow diagram of eligible studies.

This systematic review and meta-analysis were performed according to the Guidelines and checklists for Preferred Reporting Items for Systematic Review and Meta-Analysis (PRISMA) 2009 [37]. An extensive search was conducted in international databases (PubMed, ScienceDirect, Web of Science, Google Scholar, and Cochrane Library) and other sources (Google Engine and University Library Databases). Articles were searched using MeSH key terms and phrases in combination or separate using Boolean operators (“OR”/“AND”) such as “Prevalence,” “Intestinal Parasites,” “Enteric Bacterial Infections,” “Associated Factors,” “Food Handlers,” and “Ethiopia.” The study was carried out from November 2021 to June 2022. The search process was presented following PRISMA flow chart 2009 guidelines along the studies included and excluded with reasons of exclusion (Figure 1).

### 2.3. Criteria for Inclusion and Exclusion of Studies

In this systematic and meta-analysis review, institutional and hospital-based studies were included. Articles collected through the searches were evaluated for inclusion in the meta-analysis based on the following criteria: (i) Selected Ethiopian region (Amhara, Oromia, SNNPR, and Tigray) studies on the prevalence of IPs and EBIs as well as their risk factors among food handlers with a sample size of at least 100 observations; (ii) only human studies category and reported in English; (iii) cross-sectional and case-control studies; (iv) recent journals studied from 2014 to 2022; (v) only bacterial and parasitic etiological agents; and (vi) articles published and available online were included in this study. However, reports about knowledge and practice of food handlers towards IPs and EBIs, investigation on patterns of antimicrobial resistance of EBIs only, studies conducted outside selected regions and duplicate publications or extensions of analysis from original studies, as well as studies that were incompletely presented, were excluded from the review process. Four Ethiopian regions were included in the study due to their large population size, increasing urbanization and cover a wide range of geographical locations, along with many infrastructures, namely, hotels, resorts, cafeterias, and organizations (governmental and nongovernmental). Among many of the previously published articles, only 20 met the meta-analysis selection criteria (Figure 1).

### 2.4. Data Extraction

The data extraction protocol consists of the name of the country, author and year of publication, study setting, study area, sample size, number of positive cases, prevalence and their associated risk factors. If the study was conducted over a range of years, then the latest year of the stated range was used. The period from January 1 to March 30, 2022, was used for study selection, quality evaluation, and data extraction.

### 2.5. Quality Assessment of Individual Studies

The general quality of the evidence was assessed using the GRADE approach (Grading of Recommendations Assessment, Development, and Evaluation) [38]. Using the three (methodological quality, comparability, and the outcome and statistical analysis of the study) main assessment tools, the quality of each study was determined. High-quality publications received five up to six points, moderate-quality publications received four points, and low-quality articles received zero up to three points. The choice and evaluation of the articles' quality were performed independently by the two reviewers (AG and AA). The articles were added after agreement was reached and the discrepancies between the reviewers were resolved through discussion.

### 2.6. Risk of Publication Bias

Using funnel plot symmetry, Cochran's *Q* test, and the *I*^2^ test, the risks of publication bias in articles were analyzed.

### 2.7. Statistical Analysis

The pooled prevalence of IPs and EBIs among food handlers was calculated by dividing the total positive cases by the total study subjects included in this meta-analysis and multiplying by a factor of a hundred. We used a random-effects model to estimate the pooled effect size. To sort out the causes of heterogeneity, subgroup analysis was conducted based on sample size, region of the study, year of publication, and study area. The Cochran Q statistic with inverse variance and funnel plot symmetry was used to assess the existence of statistical heterogeneity. A log odds ratio was used to decide the association between IPs and EBIs with the associated risk factors among food handler respondents included in the studies. Meta-analysis was performed using Stata software version 16, where *P* ≤ 0.05 was considered statistically significant.

## 3. Results

A total of 131 articles on the prevalence and associated risk factors of IPs and EBIs among food handlers in Ethiopia were recovered. Thirty-eight of these articles were excluded due to duplicates. Of the remaining 93 articles, 54 were excluded based on specific criteria included in the inclusion criteria and the data extraction protocol. Of the remaining 39 articles, 19 articles were further excluded due to the fact that they did not have OR, 95% CI, and the number of positive cases (meaning the report was based only on the estimated prevalence percentage). Therefore, only 20 of the studies met the eligibility criteria and were included in the final systematic review and meta-analysis study (Figure 1).

The prevalence of intestinal parasites among Ethiopian food handlers were assessed from 20 eligible studies conducted from 2014 to 2022 (9 year study). Helminthes, protozoan, and enteric bacteria were the most prevalent intestinal parasites and enteric bacterial infections among Ethiopian food handlers with a pooled prevalence of 13.89%, 11.88%, and 3.39%, respectively. 7.58%, 6.78%, 3.67%, 2.78%, and 2.70% was, respectively, the pooled prevalence of *Ascaris lumbricoides*, *Entamoeba histolytica*/*dispar*, *Giardia lamblia*, *Salmonella* spp., and hookworm (Table 1).

### 3.1. Characteristics of the Eligible Studies

Table 2 presents the characteristics of the studies required for analysis. Twenty studies were eligible and thus were included in the meta-analysis. Studies were conducted between 2014 and 2022, and all of them were cross-sectional studies. Seven and 13 studies were carried out between 2014 and 2018, as well as between 2019 and 2022, respectively. Based on the criteria, four regions, namely, Tigray (3 articles), Oromia (5 articles), Amhara (6 articles), and SNNPR (6 articles) were involved. The prevalence of intestinal parasites (IPs) and enteric bacterial infections (EBIs) among eligible studies ranged from 9.9% to 61.9% (Table 2).

### 3.2. Pooled Prevalence of IPs and EBIs

**Figure 2:**
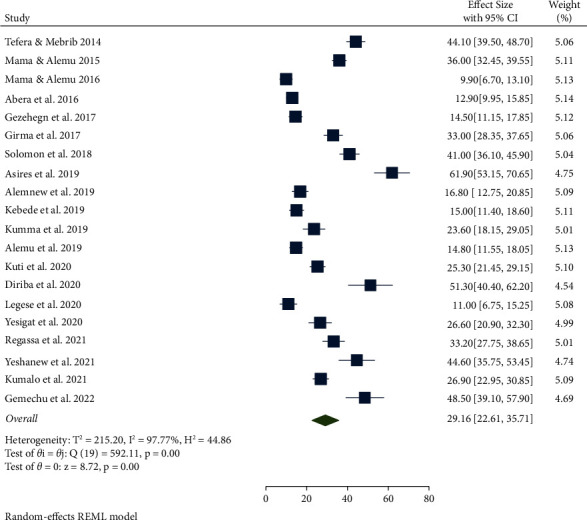
Forest plot of the pooled prevalence of IPs and EBIs among food handlers in Ethiopia from 2014 to 2022.

A random-effects model was employed to estimate the pooled prevalence of IPs and EBIs among food handlers in Ethiopia. The total national prevalence of IPs and EBIs among food handlers was 29.16 (95% CI: 22.61–35.71) (Figure 2).

#### 3.2.1. Subgroup Analysis

**Figure 3:**
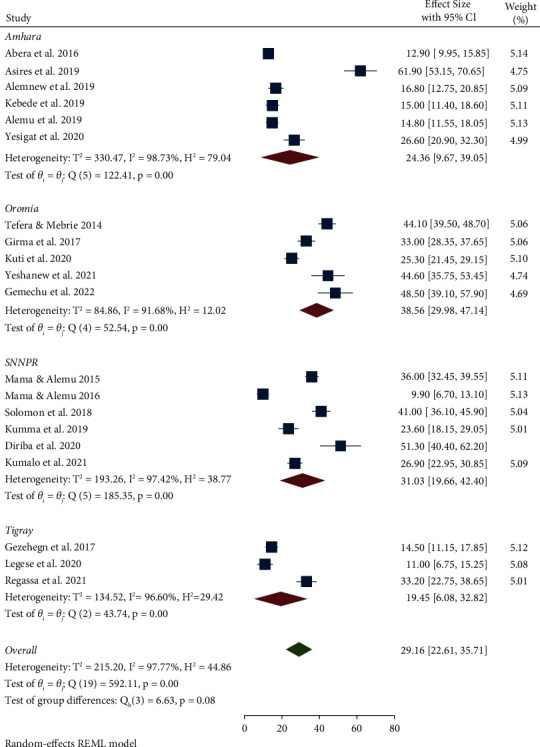
Pooled prevalence of IPs and EBIs among food handlers by region.

The high pooled prevalence of IPs and EBIs was reported from sample size <200; 36.39% (95% CI: 27.18, 45.60), followed by > 200 sample size; 27.34% (95% CI: 19.64, 35.04) (Table 3 and S1). Regarding the regional distribution, a high prevalence of IPs and EBIs among food handlers was observed in Oromia (38.56%; 95% CI: 29.98, 47.14), followed by SNNPR (31.03%; 95% CI: 19.66, 42.40), and Amhara (24.36%; 95% CI: 9.67, 29.05), while a low prevalence of IPs and EBIs was observed in the Tigray region (19.45%; 95% CI: 6.08, 32.82) (Table 3 and Figure 3). High heterogeneity in the prevalence of IPs and EBIs was observed across studies within and between three regions (Amhara, SNNPR, and Tigray) (*I*^2^ > 96% and *P* < 0.001) (Figure 3)

### 3.3. Factors Associated with IPs and EBIs among Food Handlers in Ethiopia

In this meta-analysis, food hygiene training, finger nail status, medical checkup, hand washing before food handling, hand washing before meals, and eating raw vegetables and meat were significantly associated with IPs and EBIs among food handlers (S4, S5, S6, S7, S8, and S9).

The results from five studies revealed that food hygiene training was strongly associated with IPs and EBIs. Food handlers who did not receive food hygiene training were 0.68 times more likely to have IPs and EBIs than those who had received food hygiene training (OR: 0.68, 95% CI: −0.34, 1.69, *p*=0.03) (S4).

The association between fingernail trimming and hygiene habits, as well as IPs and EBIs in Ethiopian food handlers was calculated from 15 studies. The pooled results showed that food handlers with untrimmed fingernail and poor cleanness habits were 2.23 times more likely to be infected with IPs and EBIs than their counterparts (OR: 2.23, 95% CI: 1.47, 2.99, and *p* < 0.001) (S5).

The pooled results of eight studies showed that medical checkup were strongly associated with IPs and EBIs among food handlers. The probability of IPs and EBIs was 1.52 times higher among food handlers without attending medical checkup than their counterparts (OR: 1.52, 95% CI: 0.41, 2.64, *p* < 0.001) (S6).

The odds of having IPs and EBIs were 1.97 times higher among food handlers who did not wash their hands before food handling than people who wash their hands (OR: 1.97, 95% CI: 0.53, 3.41, *p*=0.01) (S7).

The association between raw meat and vegetable eating habits with IPs and EBIs was evaluated in four studies. The odds of having IPs and EBIs were 2.63 times higher among food handlers who had habits of eating raw meat and vegetables as compared to counterparts (OR: 2.63, 95%; CI: 0.92, 4.34, *p*=0.05) (S8).

**Figure 4:**
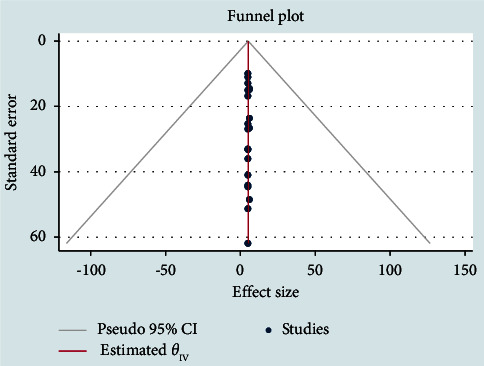
Meta funnel plot presentation, an indication of publication bias among studies in Ethiopia from 2014 to 2022.

Fourteen studies (70.0%) obtained high-quality scores, whereas six (30.0%) had medium-quality scores when it came to risk bias assessment (Table 1). The most common biases observed were representation and case definition. To know the effect of medium-quality studies, the pooled prevalence was calculated without involving it. The pooled prevalence estimates with and without these studies had confidence intervals that overlapped, indicating that there was no meaningful difference between them (Figure 4). Based on these findings, the majority of the primary study authors met high-quality standards (Figure 4). This gives the current study more credibility for healthcare providers, users, and policy makers.

## 4. Discussion

Food-borne parasitic and enteric bacterial illnesses are major public health concerns around the world, causing morbidity and mortality, especially in undeveloped countries like Ethiopia [59]. Personal sanitation and education of food handlers are as important as hygienic food preparation and delivery. This group of persons is involved in the handling, storage, transportation, processing, and preparation of food for users on a variety of levels. Knowing the exact pooled prevalence of intestinal parasites (IPs) and enteric bacterial infections (EBIs) among Ethiopian food handlers is useful as a guide for both governmental and nongovernmental policymakers and stakeholders to control food-related diseases.

The overall prevalence of IPs and EBIs among four region food handlers was 29.16% (95% CI: 22.61, 35.71) with covering (25.77%) and (3.39%) by IPs and EBIs, respectively. This 25.77% IPs prevalence was in line with findings in a previous study conducted in Ethiopia by Tegen et al. [35], who reported a prevalence of 25.01%. Similar studies by Liao et al. [60], Reh et al. [61], Harizanov et al. [62], and Staudacher et al. [63], who reported a prevalence of 28.6%, 28%, 25.53%, and 25.4% in the Democratic Republic of Congo, Spain, Bulgaria and Rwanda, respectively, were in close agreement with the current finding. However, the findings of this study was higher than those of studies conducted by Ismail, [64], 18.7% in Saudi Arabia, Coulibaly et al. [65], 18.7% in Côte d'Ivoire, Venkatajothi, [66], 17.4% in Tanzania and Abu-Madi et al. [67], 5.93% in Qatar. On the other hand, this result was lower than the reports by Sylla et al. [68], 32.6% in Senegal, Sanprasert et al. [69], 37.8% in Thailand, Al-Jawabreh et al. [70], 39.21% in Palestine, Tigabu et al. [71], 39.84% in Ethiopia, Walana et al. [72], 42.9% in Ghana, Suliman et al. [73], 54.2% in Sudan, Karshima, [74], 54.8% in Nigeria, Kimosop et al. [75], 56% in Kenya, Pasaribu et al. [76], 57.4% in Indonesia, Quihui et al. [77], 65% in Mexico, Yusuf et al. [78], 72.3% in Malaysia, Nsagha et al. [79], 74.3% in Cameroon, Erismann et al. [80], 84.7% in Burkina Faso, Osman et al. [81], 85% in Libya, and hamady obeid Al-Taei, [82], 98.8% in Iraq. This difference could be attributed to changes in hygiene and sanitation, environmental pollution, and the specificity and sensitivity of the diagnostic procedures used in various investigations.

In this study, the high pooled prevalence of IPs and EBIs among food handlers of the selected region was reported in the Oromia region at 38.56% (95% CI: 29.98, 47.14), followed by SNNPR 31.03% (95% CI: 19.66, 42.40), Amhara 24.36% (95% CI: 9.67, 29.05), and the Tigray region at 19.45% (95% CI: 6.08, 32.82). Ethiopia and Nepal both reported similar findings by Tegen et al. [35] and Chandrashekhar et al. [83], respectively. This variation within a country regions may be due to differences in study area, year of study, urbanization, food safety, and hygiene awareness and sociodemographic characteristics of the society.

In the current investigation, *Ascaris lumbricoides* was the most prevalent intestinal parasite with a pooled prevalence of 7.58%. This was consistent with the findings of a similar study conducted in South Asia [84], South Africa [85], Nigeria [86], and in different regions of Ethiopia [21, 25, 30, 87–89]. The current study found a high prevalence of *A*. *lumbricoides*, which could be attributed to the high level of environmental contamination caused by a number of infected people, the durability of *Ascaris* eggs under varying environmental conditions, the high fertility, and the sticky nature of the *Ascaris* egg shell, which aids in its attachment to human hands, fruits, and vegetables.

In this meta-analysis, the pooled prevalence of *Entamoeba histolytica*/*dispar* was 6.78%. It comparatively agrees with the reports from Mexico (5%) by Quihui et al. [77], Ethiopia (6.4%) by Yeshanew et al. [56], and Cameroon (7.3%) by Nsagha et al. [79]. However, the outcome of this investigation was higher than that of previous studies conducted by Walana et al. [72] in Ghana (0.21%), Bahrami et al. [90] in Iran (0.6%), Sanprasert et al. [69] in Thailand (0.73%), and Ismail [64] in Saudi Arabia (2%). In comparison, the findings of the current study were lower than those of previous investigations conducted in Sudan (31.2%) by Suliman et al. [73], in Côte d'Ivoire (56%) by Coulibaly et al. [65], in Burkina Faso (66.5%) by Erismann et al. [80], and in Iraq (88%) by Hamady obeid Al-Taei [82]. The variance could be attributed to the quality of the food and water of the various study locations, as well as their ambient conditions. Since *E*. *histolytica*/*dispar* is a pollutant in drinking water and food as such it can easily spread through drinking polluted water and eating contaminated vegetables and foods.

The pooled prevalence of *G*. *lamblia* in this meta-analysis was 3.67%, which is comparatively consistent with previous investigations conducted by Ismail [64] in Saudi Arabia (3%), Nsagha et al. [79] in Cameroon (3.3%), Rahi and Majeed, [91] in Iraq (4%), Sanprasert et al. [69] in Thailand (4.2%), Ghenghesh et al. [92] in Libya (4.9%), Okyay et al. [93] in Turkey (6.1%), and Kimosop et al. [75] in Kenya (6.5%). However, it was lower than in previous findings carried out in Ethiopia (10.03%) by Tegen et al. [35], Tanzania (10.6%) by Venkatajothi [66], Iraq (10.8%) by hamady obeid Al-Taei [82], Ghana (12.2%) by Walana et al. [72], Nepal (12.5%) by Erismann et al. [80], Côte d'Ivoire (13.1%) by Coulibaly et al. [65], Dhaka (17.6%) by Shahid et al. [94], Spain (18%) by Reh et al. [61], Philippines (19.2%) by Weerakoon et al. [95], Senegal (20.4%) by Sylla et al. [68], Sudan (22.9%) by Suliman et al. [73], Mexico (24%) by Quihui et al. [77], Burkina Faso (28.1%) by Erismann et al. [80], Libya (28.5%) by Osman et al. [81], Democratic Republic Congo (31.5%) by Liao et al. [60], Turkey (47.97%) by Doni et al. [96], and Bulgaria (62.05%) by Harizanov et al. [62]. However, the prevalence of *G*. *lamblia* (3.67%) in this study was significantly higher than that of Punsawad et al. [97] in Thailand (0.6%) and Bahrami et al. [90] in Iran (1.7%). The discrepancy might be attributed to educational status, insufficient sanitary surveillance by the regulatory team, and a lack of hand washing facilities in the workplace.

The pooled prevalence of hookworm was 2.70%, which is in agreement with other Ethiopian studies by Aklilu et al. [25] in Addis Ababa (2.1%) and Sahlemariam and Mekete [30] in Jimma (2.9%). The high frequency of such parasite could be attributed to poor personal hygiene and the parasite's simple mode of transmission, which is commonly found in contaminated soil, or surfaces contaminated with feces. Additionally, the disparity between studies could be explained by differences in eating habits, climate conditions, and other sociocultural variances between the location of the research and the year.

In the current study, the prevalence of *Salmonella* was (2.78%), which was comparable with a study from Motta town (2.5%) by Yesigat et al. [54], Sudan (3.8%) by Saeed and Hamid. [16], Debre Markos University (3.6%) by Mengist et al. [98] and Addis Ababa (3.8%) by Belhu et al. [34]. However, a higher prevalence of *Salmonella* was reported from Nigeria (5.5%) by Mobolaji and Olubunmi [99], Dire Dawa city (6%) by Tadesse et al. [100], Arbaminch (6.9%) by Mama and Alemu [41], and Wolyta Sodo (9.1%) by Solomon et al. [45] compared to the current finding. These differences in *Salmonella* prevalence may be related to differences in personal hygiene and geographic location.

In this finding, the pooled prevalence of *Shigella* was 0.61%, which is comparatively in line with the findings of Dire Dawa city (1.7%) by Tadesse et al. [100], Motta Town (1.6%) by Yesigat et al. [54], and Bahir Dar University (1.2%) by Abera et al. [42]. As compared to the current study, a higher prevalence of *Shigella* was reported from Gondar University (2.7%) by Dagnew et al. [101] and Gondar town (10.1%) by Getie et al. [102]. This difference could be attributed to the culture medium used and to the geographical distribution.

Food handlers who did not receive food hygiene training were 0.68 times more likely to have IPs and EBIs than those who had received food hygiene training. It is supported by other studies conducted elsewhere in Ethiopia by Abera et al. [103], Nigusse et al. [21], and Gizaw et al. [104]. This could be due to differences in the number of institutions engaged in safety, employers' proclivity to hire food handlers without considering a health certificate as a basic criterion, and low monthly wage (paid) for food handlers in other study locations.

The pooled results showed that food handlers with untrimmed fingernail and poor cleanness habits were 2.23 times more likely to be infected with IPs and EBIs than their counterparts. This finding is supported by studies conducted in Ethiopia by Tegen et al. [35], by Eshetu et al. [105] in Sri Lanka by Galgamuwa et al. [106] and in Nepal by Sah et al. [107]. Due to the difficulties of cleaning, untrimmed fingernails among food handlers could serve as a vehicle for transporting IPs and EBIs from source to food. Food handlers with untrimmed fingernails may also contaminate food while serving customers if they are infected, and they can be identified as possible public health threats. Furthermore, this may most likely owing to a lack of awareness, poor hygiene practices, and sociodemographic characteristics among the food handlers.

The probability of IPs and EBIs was 1.52 times higher among food handlers who did not attend a medical checkup than among their counterparts. It agrees with earlier studies conducted in Ethiopia by Marami et al. [29] and Gezehegn et al. [43]. Therefore, it is better Ethiopian food handlers to update their medical certificates every three months that reduces the prevalence of IPs and EBIs among them and the wide customers.

The odds of having IPs and EBIs were 1.97 times higher among food handlers who did not wash their hands before food handling with soap and water than among people who wash their hands. This finding was in line with the previous study conducted in Ethiopia by Tegen et al. [35], in Nigeria by Amuta et al. [108], in Indonesia by Pasaribu et al. [76], and in Cameroon by Tchakounté et al. [109]. This could be due to effective hand washing techniques interrupt the chain of transmission for IPs and EBIs.

The odds of having IPs and EBIs were 2.63 times higher among food handlers who had habits of eating raw meat and vegetables as compared with the counterparts. It agrees with the findings conducted in Ethiopia by Tolera and Dufera [110] and Alemu et al. [111] and outside in Sri Lanka by Galgamuwa et al. [106] and in Libya by Osman et al. [81]. This could be due to raw meat and unwashed vegetables can carry IPs and EBIs causing pathogens.

### 4.1. Limitation of the Study

A small number of published papers met the inclusion criteria and were involved in the current finding.

## 5. Conclusion

The pooled prevalence of IPs and EBIs was 29.16%. *Ascaris lumbricoides*, *Entamoeba histolytica*/*dispar*, *Giardia lamblia*, and hookworm were the most prevalent IPs among food handlers with a pooled prevalence of 7.58%, 6.78%, 3.67%, and 2.70%, respectively. *Salmonella* and *Shigella* spp. were the most prevalent EBIs among Ethiopian food handlers with a pooled prevalence of 2.78% and 0.61%, respectively. Food handlers who did not receive food hygiene training, untrimmed finger nail, eating raw vegetables and meat, lack of periodic medical checkup and hand washing habits were factors significantly associated with the prevalence of IPs and EBIs. Increasing food handler's knowledge about personal hygienic conditions and periodic medical checkup for IPs and EBIs could consider an appropriate intervention measure.

## Figures and Tables

**Table 1:** Prevalence of intestinal parasites and enteric bacteria among food handlers.

Category	Pooled number (%)
Helminthes	724 (13.89)
*Ascaris lumbricoides*	395 (7.58)
Hookworm	141 (2.70)
*Taenia* species	58 (1.11)
*Trichuris trichiura*	53 (1.02)
*Enterobius vermicularis*	29 (0.56)
*Schistosoma mansoni*	26 (0.50)
*Hymenolepis nana*	22 (0.42)

Protozoan	619 (11.88)
*Entamoeba histolytica*/*dispar*	353 (6.78)
*Giardia lamblia*	191 (3.67)
*Entamoeba coli*	50 (0.96)
*Entamoeba hartmanni*	18 (0.34)
*Giardia intestinalis*	7 (0.13)

Enteric bacteria	177 (3.39)
*Salmonella* spp.	145 (2.78)
*Shigella* spp.	32 (0.61)
Total	1,520 (29.16)

**Table 2:** List and characteristics of 20 eligible studies conducted from 2014 to 2022 among food handlers.

Authors	Year	Region	Study Area	Sample Size	Case	Prevalence (95% CI)	Quality Score
Tefera and mebrie	2014 [39]	Oromia	Yebu town	118	52	44.1 (36.6–45.8)	4
Mama and alemu	2015 [40]	SNNPR	Arba minch university	345	123	36.0 (32.7–39.8)	4
Mama and alemu	2016 [41]	SNNPR	Arba minch university	345	34	9.9 (6.3–12.7)	4
Abera et al.	2016 [42]	Amhara	Bahir dar university	410	53	12.9 (9.8–15.7)	4
Gezehegn et al.	2017 [43]	Tigray	Aksum town	400	58	14.5 (11.3–18.0)	6
Girma et al.	2017 [44]	Oromia	Jimma university	148	31	33.0 (28.2–37.5)	5
Solomon et al.	2018 [45]	SNNPR	Wolaita sodo town	387	159	41.0 (35.4–45.2)	5
Asires et al.	2019 [46]	Amhara	East and west gojjam	416	213	61.9 (51.2–68.7)	4
Alemnew et al.	2019 [47]	Amhara	Woldia university	256	43	16.8 (13.1–21.2)	5
Kebede et al.	2019 [48]	Amhara	Wollo university	200	30	15.0 (12.1–19.3)	5
Kumma et al.	2019 [49]	SNNPR	Wolaita sodo university	233	55	23.6 (18.2–29.1)	6
Alemu et al.	2019 [50]	Amhara	Chagni town	422	62	14.8 (11.5–18.0)	6
Kuti et al.	2020 [51]	Oromia	Madda walabu university	198	50	25.3 (21.9–29.6)	5
Diriba et al.	2020 [52]	SNNPR	Dilla university	220	113	51.3 (40.9–62.7)	4
Legese et al.	2020 [53]	Tigray	Adigrat university	301	33	11.0 (6.1–14.6)	5
Yesigat et al.	2020 [54]	Amhara	Motta town	243	67	26.6 (21.5–32.9)	6
Regassa et al.	2021 [55]	Tigray	Medebay zana district	401	129	33.2 (27.3–38.2)	5
Yeshanew et al.	2021 [56]	Oromia	Mettu town	139	62	44.6 (34.6–52.3)	5
Kumalo et al.	2021 [57]	SNNPR	Dawuro zone	402	108	26.9 (22.7–30.6)	5
Gemechu et al.	2022 [58]	Oromia	Jimma town	260	125	48.5 (38.7–57.5)	6

**Table 3:** Prevalence of IPs and EBIs among food handlers in Ethiopia by subgroup analysis.

Variables	Characteristics	Number of studies	Sample size	Prevalence(95% CI)	*I* ^2^, *P* value
Sample size	<200	4	603	36.39 (95% CI: 27.18, 45.60)	92.56%, *P* < 0.001
	>200	16	5241	27.34 (95% CI: 19.64, 35.04)	98.07%, *P* < 0.001

Pooled prevalence of IPs and EBIs by region	Amhara	6	1947	24.36 (95% CI: 9.67, 39.65)	98.73%, *P* < 0.001
	Oromia	5	863	38.56 (95% CI: 29.98, 47.14)	91.68%, *P* < 0.001
	SNNPR	6	1932	31.03 (95% CI: 19.66, 42.40)	97.42%, *P* < 0.001
	Tigray	3	1102	19.45 (95% CI: 6.08, 32.83)	96.60%, *P* < 0.001

Pooled prevalence of IPs and EBIs by year	2014–2018	7	2153	27.26 (95% CI: 16.55, 37.97)	98.27%, *P* < 0.001
	2019–2022	14	3691	30.16 (95% CI: 21.68, 38.83)	97.46%, *P* < 0.001

Pooled prevalence of IPs and EBIs by study area	University	10	2656	23.04 (95% CI: 15.24, 30.84)	97.34%, *P* < 0.001
	Town/district/zone	10	3188	35.25 (95% CI: 25.87, 44.63)	97.23%, *P* < 0.001

*Overall*		20	5844	29.16 (95% CI: 22.61, 35.71)	97.77%, *P* < 0.001

## Data Availability

All data sets have been presented within the manuscript and on supplementary data. The dataset supporting the conclusions of this article is available from the correspondence author upon a formal request.

## Abbreviations

AOR: Adjusted odds ratio
CI: Confidence interval
EBIs: Enteric bacterial infections
GRADE: Grading of Recommendations Assessment, Development and Evaluation
IPs: Intestinal parasites
PRISMA: Preferred Reporting Items for Systematic Reviews and Meta-Analyses
SNNPR: Southern Nations, Nationalities, and Peoples' Region
STATA: Statistical software for data science.

## Supplementary Materials

Supplementary Materials S1: pooled prevalence of IPs and EBIs among food handlers by sample size. S2: pooled prevalence of IPs and EBIs among food handlers from 2014 to 2022. S3: pooled prevalence of IPs and EBIs among food handlers by study area. S4: food hygiene training as an associated risk factor for IPs and EBIs among food handlers. S5: fingers nail status as an associated risk factor for IPs and EBIs among food handlers. S6: medical checkup as an associated risk factor for IPs and EBIs among food handlers. S7: hand washing habit before food handling as an associated risk factor for IPs and EBIs among food handlers. S8: eating raw vegetables and meat as an associated risk factor for IPs and EBIs among food handlers.
