# Supplementary material for: Prevalence and Associated Risk Factors of Intestinal Parasites and Enteric Bacterial Infections among Selected Region Food Handlers of Ethiopia during 2014–2022: A Systematic Review and Meta-Analysis
Source: ScientificWorldJournal. 2022 Oct 12;2022:7786036. doi: 10.1155/2022/7786036 (PMC9581692; doi:10.1155/2022/7786036)
Supplement: Supplementary Materials — S1: pooled prevalence of IPs and EBIs among food handlers by sample size. S2: pooled prevalence of IPs and EBIs among food handlers from 2014 to 2022. S3: pooled prevalence of IPs and EBIs among food handlers by study area. S4: food hygiene training as an associated risk factor for IPs and EBIs among food handlers. S5: fingers nail status as an associated risk factor for IPs and EBIs among food handlers. S6: medical checkup as an associated risk factor for IPs and EBIs among food handlers. S7: hand washing habit before food handling as an associated risk factor for IPs and EBIs among food handlers. S8: eating raw vegetables and meat as an associated risk factor for IPs and EBIs among food handlers. [file 7786036.f1.zip › S7..pdf]

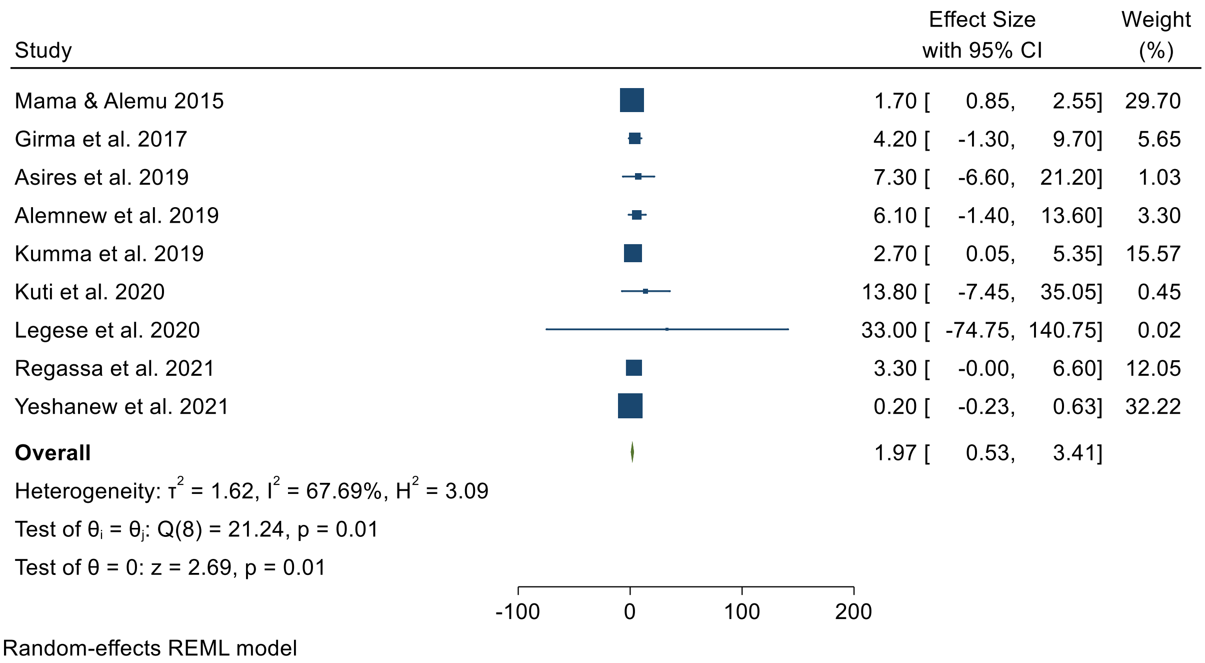

S7: Hand washing habit before food handling as an associated risk factor for IPs and EBIs among food handlers
